# Peptide-Based Drugs and Drug Delivery Systems

**DOI:** 10.3390/molecules22122185

**Published:** 2017-12-08

**Authors:** Stefania Galdiero, Paula A. C. Gomes

**Affiliations:** 1 Department of Pharmacy, School of Medicine, University of Naples Federico II, Via Mezzocannone 16, 80134 Naples, Italy; 2 Interuniversity Research Centre on Bioactive Peptides (CIRPEB), University of Naples Federico II, Via Mezzocannone 16, 80134 Naples, Italy; 3 LAQV-REQUIMTE, Department of Chemistry and Biochemistry, Faculty of Sciences, University of Porto, Rua do Campo Alegre 687, 4169-007 Porto, Portugal

It is undoubtable that the scientific community is devoting a great deal of attention and resources to peptides, while the whole world is keeping track of their prospective therapeutic and biomedical applications. In fact, by virtue of its topic, this special issue has attracted much interest from leading scientists in the field, which is demonstrated by the 23 high-quality contributions received, 15 of which reported cutting-edge original research, with the remaining 8 being literature reviews of clear timeliness. This growing focus on peptide therapeutics is not a mere matter of coincidence or fashion; indeed, a deeper analysis of publications in this issue shows that we are witnessing an enormous interest in antimicrobial peptides (AMPs), with a slightly lower percentage of articles tackling cancer and drug delivery, which is in line with the growing focus of recent peptide-related literature on AMPs.

The regained and growing interest in AMPs is intimately related to antibiotic resistance, which represents a terrible economic, social and health burden worldwide, and is a major threat for mankind in the near future, as recently recognized by the World Health Organization (updated key facts on antimicrobial resistance accessible through http://www.who.int/mediacentre/factsheets/fs194/en/). The focus in the literature on AMP—and this special issue is no exception—forecasts the importance of developing new effective therapeutic agents for a post-antibiotic era. In this context, AMPs represent a potential alternative to be considered for fighting infectious diseases, thanks to their capacity for acting on multiple targets, at low dose, and by means of a variety of mechanisms, which makes the induction of resistance less likely [1,2]. The current challenge is to translate results from in vitro studies into clinical practice. Papers published in this issue address many relevant aspects in this topic, from discovery to activity, as follows.

New insight into the identification and characterization of novel AMPs is reported by Al Akeel et al. [3]. New promising anionic antimicrobial compounds were derived from *Foeniculum vulgare*, a therapeutic plant belonging to the *Umbelliferae* (Apiaceae) family, which is widely used in traditional chinese medicine.

Silva et al. [4] nicely review the immunomodulatory activity of AMPs and their role in host defense with a main focus on intra-macrophagic pathogens. They have mainly addressed cathelicidins and defensins as prototypic host defense peptides, and knowledge of the immunomodulatory effects of these AMPs is central to enhance their antimicrobial activity and chiefly to anticipate possible immune-related negative effects.

Pero et al. [5] discuss the potential use of AMPs, either alone or in combination with conventional antibiotics, for the treatment of *Helicobacter pylori* (Hp) infections in humans. They mainly focus on the role played by human β-defensins to fight Hp, which infects about 60% of adults worldwide, is responsible for most gastric and duodenal ulcers, and has been considered a major causative agent of gastric cancer, the fifth most common cancer in the world, with the third highest mortality rate.

An increasing number of papers in recent literature concerns the potential interest of AMPs and other peptides in tackling problems related to wound healing/regeneration [6]. Gomes et al. [7] have focused on peptides whose wound-healing effects may find application in the topical treatment of diabetic foot ulcers, as well as other skin and soft tissue infections, holding great promise for development of novel strategies for treatment of chronically infected wounds. The authors described the applications of several AMPs of human and amphibian origin, which can influence different mechanisms of the wound-healing process, such as inflammation, epithelialization, tissue granulation and remodeling.

In the frame of developing AMPs as alternatives to antibiotics, one of the main challenges is related to problems such as the high costs of peptide production, low in vivo stability, and eventual presence of side effects. Rational design appears to be of chief importance for maintaining the crucial features of native AMPs while enhancing their activity and displaying extended stability; in fact, several works face this issue by means of developing antimicrobial peptidomimetics. Molchanova et al. [8] provide a critical overview of major holdups towards therapeutic application of AMPs, among which is their short plasma half-life. Recent advances in the development of antimicrobial peptidomimetics as potential drugs are discussed with respect to accessibility via chemical synthesis, as well as optimization strategies for obtaining compounds with a relevant pharmacological profile. This paper is a comprehensive review addressing key issues such as availability, toxicity, bioavailability, and approval regulations. 

Falanga et al. [9] addressed the same issue in a review focused on the higher serum stability of cyclic peptides as a means to design novel antimicrobial agents. This contribution has a particular focus on θ-defensins, whose scaffold is very different from those of α-defensins and β-defensins, and was exploited as a leading synthetic structure for the development of novel cyclic antibacterial compounds with higher stability as compared to linear sequences, while having low synthetic challenge and cost.

Cirac et al. [10] were interested in the design of novel AMPs for the treatment of plant diseases in order to substitute the main agents used in crop protection, such as copper derivatives and antibiotics, which are environmental contaminants and may cause development of resistance. The authors employed molecular dynamics (MD) simulations as a tool to recognize the factors that drive formation of stable, partially amphipathic β-structures in cyclic peptides with antimicrobial activity.

Interestingly, long after their discovery, lactoferricins remain a hot topic in the field of AMPs; for instance, bovine lactoferricin, a 25-amino acid AMP obtained from bovine lactoferrin protein, which has antiviral, antiparasitic, antifungal, anticancer, and antibacterial activity against Gram-positive and Gram-negative bacteria, served as the inspiration for a couple of papers published by the group of Castañeda in this special issue. In a first paper, they reported that synthetic peptides containing the RRWQWR sequence exhibited activity similar to or greater than the native sequence [11]; while in the second paper, they showed that the monomeric, cyclic, tetrameric, and palindromic peptides containing this motif exhibited high and specific activity against *E. coli* [12].

Another challenging approach is conjugation of AMPs to nanoparticles (NPs), as reported by Libralato et al. [13]. This allows the targeting of infected cells, improving antimicrobial activity and decreasing side effects, while making necessary the assessment of NP-associated toxicity issues. As such, this review focuses on the toxicity of functionalized NPs based on some key bioindicators, suggesting that both long-term exposure (chronic stress) and low-level exposure may be relevant in obtaining a more generalized view of the effects of peptide-NP conjugates towards ecosystems.

Interest in early-stage diagnosis of bacterial infections is stimulated by the need for effective prevention and treatment measures; in this scenario, the development of bacteria-specific tracers is challenging, and may lead to more reliable tools for infection imaging, such as radiolabeled antibiotics, vitamins, and AMPs. In particular, the interaction of AMPs with bacterial membranes may be exploited to design imaging agents for targeting bacterial infection. Ebenhan et al. [14] report their pioneering study in this particular topic, by using a radiolabeled depsipeptide conjugate to explore its potential as a radiodiagnostic agent for the imaging of infection.

Still related with search of novel peptide-based strategies against infection, special attention was given to another important field of research; that is, the development of antiviral peptides. Peptides corresponding to heptad repeat domains of viral glycoproteins have been shown to possess antiviral activity, which can be enhanced by conjugating them to lipids, thus targeting them to the plasma membrane, where fusion occurs. Augusto et al. [15] provided new insight into the understanding of the mechanism of antiviral lipopeptides derived from the *C*-terminal heptad repeat of *Paramyxovirus* F protein. Among them, the peptide with a 24-unit PEG linker was conjugated to a cholesterol moiety, and was found to be the best membrane fusion inhibitory peptide; it also displayed a high affinity towards human peripheral blood mononuclear cells (PBMC), which could be used as an effective spreading vehicle of respiratory viruses traveling within the host to infect new tissues. Augusto et al. address the interaction of the same peptides with PBMC as a model for what may happen in the circulation. To identify lipid modifications that would retain the anchoring property provided by cholesterol while increasing the dynamic nature of membrane interactions, Gomes et al. [16] reported on *C*-terminal heptad repeat (HRC) peptides derived from the fusion envelope glycoprotein (F) of *Measles virus* (MV) and conjugated to 25-hydroxycholesterol (25HC) without a PEG linker. New promising antiviral candidates emerged, suggesting that the increased membrane interaction dynamicity obtained from 25HC conjugation also enhanced antiviral efficacy.

Drug delivery and target specificity is a challenging topic, because crossing of cell membranes is key to improving both therapy and diagnostics [17,18]. In this connection, Kalafatovic and Giralt [19] published a nice and comprehensive review on the scientific literature concerning cell-penetrating peptides (CPPs) and design approaches mimicking the natural viral penetration domains. A variety of CPP designs are described, such as linear and flexible sequences, positively charged, amphipathic, but also more rigid templates comprising cyclic, stapled, or dimeric and/or multivalent, self-assembled peptides or peptidomimetics. This review addresses new concepts and strategies through which the rational design of CPPs can be ameliorated to enhance the efficiency of internalization and to regulate the kinetics of this process, which may help in the transition of CPPs from bench to a clinical setting. Finding new effective shuttles able to translocate the blood-brain barrier (BBB) with no significant cellular damage is even more challenging. The capacity to transport therapeutic molecules across the BBB represents a breakthrough in the development of tools for the treatment of many central nervous system (CNS)-associated diseases and in fact, not all efficient CPPs are BBB-translocators. Interestingly, Neves-Coelho et al. [20] describe the first anionic trans-BBB peptide with negligible effects on barrier integrity; this implies a reconsideration of the role of electrostatic attraction in BBB translocation, as cationic character is no longer mandatory for a CPP to be internalized by target cells. 

Research in the field of self-assembling peptides is also attracting great attention in the scientific community [21,22]. Here, Folchman-Wagner et al. [23] reported on the use of polyelectrolyte complexes (PECs), which are structures that form spontaneously upon the combination of oppositely charged macromolecules in solution, for appealing drug-delivery applications presenting several advantages over traditional NP carriers, including controllable size, biodegradability, biocompatibility, and lack of toxicity. The authors nicely discussed the potential application of PECs for drug delivery to the slightly acidic tumor microenvironment, taking advantage of the pH-dependent ionization of various amino acid side groups. The chemotherapeutic drug daunomycin was also added to the PEC as proof of concept for utility of PECs as a drug delivery tools.

As previously mentioned, this issue also included very interesting contributions regarding the potential role of peptides against cancer. While there is global awareness on the burden of cancer to the modern society, it is relevant to add that future lack of effective antimicrobial strategies will also have an impact on anti-cancer approaches, which often involve surgical and/or immunosuppressive interventions. Cancer results from malfunction of fundamental cellular processes including cellular growth, proliferation, survival and metabolism. In this sense, natural molecules with antiproliferative activity are considered a scientific field of major interest, in order to avoid the collateral damage associated with radiation and chemotherapy. Hernández-Padilla et al. [24] reported on the antiproliferative effect of cyclic peptides or derived diketopiperazines of microbial origin, which are believed to have strong pharmaceutical potential as antimicrobial and antifungal agents, immunomodulators, antioxidants, and anticancer agents.

Peptides can also have a role for the specific targeting of receptors that are overexpressed in cancer cells. For instance, the melanocortin receptor 1 (MC1R), which belongs to a five-member subfamily of G protein-coupled receptors, is expressed in melanocytes, melanoma cells, macrophages, and brain, as well as in leukocytes, where it may mediate an anti-inflammatory action. Morais et al. [25] reported on linear and cyclic analogues of the α-melanocyte stimulating hormone (α-MSH) that specifically target MC1R, thus making it of pharmacological interest for detecting and treating melanoma.

Along the same lines, new insight into the most recent advances in chemical modifications of amino acid sequences, linkers and chelators to produce optimal moieties for diagnosis and therapy of human neoplastic diseases was provided by the valuable review of Tornesello et al. [26]. Many synthetic peptides developed for diagnosis and therapy of human cancers based on their ability to target specific receptors overexpressed on cancer cell surface or to penetrate the cell membrane are reviewed. Chemical modifications of amino acid chains have significantly improved the biological activity, the stability and efficacy of peptide analogues currently employed as anticancer drugs or as molecular imaging tracers. This review describes the most important and recent results which have significantly improved the specificity and sensitivity of peptides used in oncologic diagnosis and therapy.

Oxidative stress is related to some physiological and pathological processes, and plays important roles in cancer and many other diseases. Glutathione peroxidase (GPx) is an important antioxidant enzyme and considerable efforts have been made to develop GPx mimics. Yin et al. [27] reported on the design and activity of short 5-mer peptides (5P) effective in the treatment of liver cancer in vitro and in vivo. In summary, they showed that 5P could overcome many of the limitations of traditional anticancer drugs, and could be used at low doses to inhibit tumor growth.

Also related to peptide-based cancer approaches, a nice method for improving both oral bioavailability and drug delivery to the tumor site, and/or for minimizing toxic side effects, is the use of prodrugs. Tsume et al. [28] adopted a prodrug approach for gemcitabine with stereospecific, l-/d-amino acids, and the dipeptide l-phenylalanyl-l-tyrosine in surrogate cell systems, including the stromal environment.

Although the aforementioned works address the most popular and well-known use of peptide drugs, this special issue also comprises reports focused on other applications in health care. One example concerns control of hypertension, a major risk factor for cardiovascular and renal diseases; Li et al. [29] reported on the development of potent anti-hypertensive peptides from *Coix glutelin*, which could be a good natural ingredient for pharmaceuticals against hypertension and related diseases.

Finally, as stressed before, a very important part of the drug development process is a clear transfer from in vitro to in vivo models; striking in vitro effects are no guarantee that these will translate into comparable therapeutic activity in an in vivo experimental model. In this connection, and using a novel drug discovery technology, New et al. [30] created cyclic peptides that are able to down-regulate secretion of inflammatory cytokines both in vitro and in vivo. This holds promise against inflammation-related diseases, such as rheumatoid arthritis, on which cytokines are strongly implicated.

In conclusion, this Molecules Special Issue gathers reports on some recent advances in peptide-based drugs and drug-delivery systems, which clearly demonstrate the emerging trends in a field that will probably influence the world in the future. Huge research efforts are being invested in the discovery of novel therapeutic applications, making the future of peptide science quite promising.
